# Diflunisal therapy for cardiac ATTR amyloidosis: a longitudinal, prospective, single centre study

**DOI:** 10.1186/1750-1172-10-S1-O23

**Published:** 2015-11-02

**Authors:** Candida C Quarta, Sevda Ozer, Carol J Whelan, Marianna Fontana, Dorota M Rowczenio, Janet A Gilbertson, Philip N Hawkins, Julian D Gillmore

**Affiliations:** 1 Royal Free Hospital, Division of Medicine, University College of London, National Amyloidosis Centre, NW3 2PF, London, UK

## Background

The transthyretin (TTR) stabilizer diflunisal has been shown to reduce the rate of progression of neurological manifestations in patients with hereditary ATTR amyloidosis. However, data on the effect of diflunisal on cardiac structure and function in amyloidotic hearts are lacking. We report the echocardiographic profiles and cardiac biomarkers of patients with either mutant (M) or wild-type (WT) TTR-related cardiac amyloidosis (ATTR-CA) who received treatment with diflunisal compared to matched untreated patients.

## Methods

We included in the analysis patients with clearly defined ATTR-CA, who received diflunisal for at least 24 months. For comparison, we included a group of patients with similar age, left ventricular (LV) wall thickness and renal function who were not treated with diflunisal. Patients with coexistent rhythm abnormalities (e.g. atrial fibrillation) or pacemaker implantation were excluded. All subjects underwent a standardized comprehensive protocol of evaluations and follow-up.

## Results

We identified 18 patients aged 70 [67-73] who received diflunisal for at least 24 months (9 WT; 9 M: 7 T60A, 1 S77Y, 1 G47R). For comparison we included 17 untreated patients, aged 70 [68-75] (p=0.43) (14 WT; 3 M: 2 T60A, 1 V122I). At baseline, treated and untreated patients did not show significant differences in terms of LV wall thickness (16.6±2 vs. 16.9±2 mm, p=0.45), LV ejection fraction (53±9 vs. 48±10 %, p=0.1), and global longitudinal strain (GLS, -11.4±4 vs. -10.9±4%, p=0.74). NT-proBNP (log transformed) was 5.9±0.9 and 6.2±0.7ng/L in the treated and untreated group respectively (p=0.41). Estimated glomerular filtration rate (eGFR) was mildly reduced in both treated and untreated patients (69±2 vs. 63±20 ml/min, p=0.3).

Over 42 [34-64] months, LV wall thickness remained stable and comparable in both groups (p=0.76). LV ejection fraction worsened within the untreated group (p=0.03), but remained stable in the treated one (p=0.2), although there was no significant difference in rate of decline between the groups (p=0.24). GLS worsened in the untreated group (from -10.9±4 to -8.7±3 %, p=0.03) but not in the treated one (from -11.4±4 to -11.2±3 %, p=0.88; generalized estimating equation p=0.02). NT-proBNP increased in both the untreated group (from 6.2±0.7 to 6.9±0.6 ng/L, p<0.001) and (less) in the treated one (from 5.9±0.9 to 6.2±0.7 ng/L, p=0.02; generalized estimating equation p<0.01), in the absence of a significant decrease of eGFR over time, both between groups (generalized estimating equation p=0.29) and within each group (from 69±17 to 65±20 ml/min in the treated group, p=0.19; from 63±20 to 55±22 ml/min in the untreated group, p=0.07).

## Conclusion

The use of sensitive markers of change in cardiac function, such as GLS and NT-proBNP, suggests that diflunisal may slow or halt disease progression in mutant and wild-type ATTR-CA. These findings encourage further systematic study of diflunisal in ATTR-CA.

